# Must Epidemiologically Impactful Vector Control Interventions Disrupt Mosquito Population Structure? A Case Study of a Cluster‐Randomised Controlled Trial

**DOI:** 10.1111/eva.70173

**Published:** 2025-10-27

**Authors:** Tristan P. W. Dennis, W. Moussa Guelbeogo, Heather M. Ferguson, Steve Lindsay, Sagnon N'Fale, Patricia Pignatelli, Hilary Ranson, Antoine Sanou, Alfred Tiono, David Weetman, Mafalda Viana

**Affiliations:** ^1^ School of Biodiversity, One Health, and Veterinary Medicine University of Glasgow Glasgow UK; ^2^ Department of Vector Biology Liverpool School of Tropical Medicine Liverpool UK; ^3^ Centre National de Recherche et de Formation sur le Paludisme Ouagadougou Burkina Faso; ^4^ Université Joseph Ki‐Zerbo Ouagadougou Burkina Faso; ^5^ Department of Biosciences Durham University Durham UK; ^6^ Université Yembila Abdoulaye Toguyeni Fada N'Gourma Burkina Faso; ^7^ Groupe de Recherche Action en Santé Ouagadougou Burkina Faso

**Keywords:** cluster‐randomised controlled trial, disease vectors, epidemiology, genetics, malaria, vector populations

## Abstract

Large epidemiological impacts resulting from disease vector control interventions are typically associated with significant disruption of vector populations. While vector density is a frequently measured response, impacts on demography and connectivity are suspected but rarely quantified. We analysed low‐coverage whole‐genome sequence data of 893 *Anopheles gambiae* mosquitoes collected between 2014 and 2015 during a cluster‐randomized control trial (cRCT) in Burkina Faso to compare a pyrethroid‐only net (ITN) with a pyrethroid‐pyriproxyfen (ITN‐PPF) net. Despite reductions of clinical malaria by 12% and vector density by 22% in the ITN‐PPF arm, we found no significant changes in *An. gambiae* population genetic structure or diversity. We found remarkably low population differentiation and a lack of discernible clustering by treatment, time, or space. Nucleotide diversity and inbreeding coefficient remained stable between treatments, and genome‐wide scans showed no putative signatures of selection between trial arms. These results show that ITN‐PPF did not alter *An. gambiae* genetic structure, possibly due to large, vagile populations in West Africa. More widely, this is the first evidence that epidemiologically meaningful reductions in vector density may not impact genetic diversity or connectivity and challenges what constitutes adequate vector control in large populations.

## 1 Introduction

Vector control is the cornerstone of the public health response to many vector‐borne diseases (VBD). Effectiveness is typically assessed by the epidemiological impacts vector control generates, such as reduction in the prevalence or incidence of infection (Ashton et al. 2020; Yé et al. 2017). These epidemiological impacts are tied to reductions in human exposure to vector populations (Ross 1911), which can arise through reductions in vector population size, adult mosquito survival, or infection rates. Notable examples of vector control strategies that have large epidemiological impacts include the deployment of spatial repellents that reduced arboviral infection by 34.1% and *Aedes* mosquito abundance by 38.6% in Peru (Morrison et al. 2022) and the release of *Wolbachia*‐infected *Aedes aegypti* that resulted in a 78% reduction in dengue incidence in a trial in Indonesia (Indriani et al. 2023). For malaria, the deadliest VBD, responsible for over 600,000 deaths annually (World Health Organization 2023), the effects of vector control have been profound, with a 40% reduction in clinical disease incidence across sub‐Saharan Africa between 2000 and 2015 (Bhatt et al. 2015) as a result of mass insecticide‐treated bednet (ITN) distribution. Successful malaria vector control has been associated with broader changes in mosquito vector demography and ecology, including changes in biting and resting behaviour (Gatton et al. 2013; Sanou et al. 2021), species composition (Russell et al. 2011) and the emergence of insecticide resistance (Hemingway and Ranson 2000). For example, vector control has reduced *Anopheles gambiae* populations over time in East Africa, leading to *An. arabiensis* and *An. funestus* replacing it as the dominant vector of malaria (Bayoh et al. 2010; Msugupakulya et al. 2023). These examples suggest that large epidemiological impacts are linked to, and may typically require, significant perturbation of mosquito vector populations, including reducing population size (Magesa et al. 1991) or fragmenting population structure (Gimnig et al. 2003). However, the demographic effects of interventions on vector populations are difficult to measure directly and hence remain elusive and rarely recorded.

Insect populations, such as butterflies (Hanski 1998, 2011; Fountain et al. 2016; Hanski et al. 2017) and gypsy moths (Elkinton and Liebhold 1990; Kuussaari et al. 1998), have provided notable case studies of how populations are regulated, maintained, and structured, as well as how population fragmentation can lead to population declines by breaking connections among subpopulations (Fountain et al. 2016; Nieminen et al. 2001; Saccheri et al. 1998; Benedick et al. 2007). However, the impact of vector control on mosquito population dynamics, in particular population structure, remains poorly understood. Vector control may change mosquito population structure by reducing local population size below the threshold for persistence, and in doing so, compromise metapopulation viability or population fitness by driving down diversity. In contrast, in the presence of migration or strong density‐dependent regulation, local mosquito populations may persist even in the face of substantial reduction due to vector control (Brady et al. 2015; Killeen et al. 2013). It is thus important to understand the mechanisms underlying population resilience and the magnitude of reduction required to achieve an epidemiological benefit to improve the design of vector control programmes.

Changes in population size and structure leave genomic signatures in populations (Bradburd and Ralph 2019). Reductions in nucleotide diversity (π and *θw*) and increases in individual inbreeding (*F*_IS_) are often associated with population decline, which in turn is linked to a greater risk of stochastic extinction (Nieminen et al. 2001; Saccheri et al. 1998; Söderquist et al. 2020). Furthermore, changes in rates of dispersal between subpopulations may affect metapopulation viability. Inference of close‐kin relationships using genetic techniques (close‐kin mark‐recapture (CKMR)) has been used to quantify *Aedes* mosquito dispersal (Jasper et al. 2022), but is currently untested in estimating *Anopheles* taxa. Population genetics has also been used previously to estimate vector control impacts. Notable examples include a study in Kilifi, Kenya, which documented genetic signatures of population decline in *An. gambiae* following large‐scale ITN deployment from 2006. Reductions in nucleotide diversity (~5% lower π and ~15% lower *θw*), and a deficit in the number of low‐frequency sites (negative Tajima's D) were accompanied by changes in vector species composition, whereby the presence of the primary malaria vector species, *An. gambiae sensu stricto* declined from 79% of *An. gambiae* species complex samples between 1997 and 1998 to an undetectable level in 2007–2008 (O'Loughlin et al. 2016). In a separate study in western Kenya, the proportion of *An. gambiae* adults collected in surveillance dropped from 85% to 1% between 1999 and 2010, coincident with high ITN coverage (Bayoh et al. 2010), with genomic signatures of population decline such as increased inbreeding, decreased genetic diversity, and allele‐frequency shifts, evident in samples sequenced in a separate study (Miles et al. 2017). There are also reported instances where vector control was not associated with significant signatures of population disruption. In Cameroon, ITN implementation was associated with insignificant impacts on *An. arabiensis* genetic diversity and population structure (Wondji et al. 2005), though no clinical or entomological endpoints were reported with these data. Evidence of changes to population structure in the face of successful vector control is currently limited and variable.

Significant epidemiological impacts in cluster‐randomised controlled trials (cRCT) of vector control are often accompanied by declines in vector population densities. However, the impact on population structure (e.g., reductions in diversity and increases in inbreeding as a result of changes in population size, changes in between‐population differentiation as a consequence of population fragmentation or extinction and recolonisation) and the extent to which epidemiological impact requires population disruption remain unclear. This lack of clarity could stem from the lack, to our knowledge, of published studies with systematic comparisons between treatment versus control groups that also incorporate population genetic estimations of changes in structuring or diversity. This knowledge gap can be addressed by applying population genomic techniques to dense vector sampling from clinical trials, especially field cRCTs comparing control and treatment groups. Low‐coverage whole‐genome‐sequencing (WGS) approaches (e.g., a depth‐of‐coverage of < 10× compared to 30× coverage) are particularly attractive for accurate inference of population genetic parameters, such as differentiation, relatedness, inbreeding coefficient, and allele‐frequency estimation (Lou et al. 2021; Hanghøj et al. 2019; Vieira et al. 2013; Dennis et al. 2024; World Health Organization 2024). lcWGS typically relies on probabilistic models applied to genotype‐likelihoods inferred from read alignments (as opposed to called genotypes), and is robust to extremely low depths of coverage (e.g., 1× vs. 30×) with accuracy increasing with sample size (Lou et al. 2021; Hanghøj et al. 2019; Vieira et al. 2013; Dennis et al. 2024). lcWGS is appealing because it facilitates greater population sampling for a fraction of the cost of full‐coverage approaches.

Validation of new vector control strategies, such as gene‐drive approaches, spatial repellents, and tools using novel insecticide classes with different entomological modes of action often requires evaluation in cRCTs to determine their public health value. While standard guidelines are in place for the measurement of entomological and epidemiological endpoints (World Health Organization 2024), methodologies to understand how these interventions impact vector populations more holistically are missing. Population genetic analysis of vectors coupled with cRCTs can provide more information on the effect of vector control on mosquito population dynamics and, in turn, the extent to which epidemiological outcomes are coupled with substantial demographic impacts on vector populations.

Here, we use the case study of the AvecNet cRCT performed in Burkina Faso (Tiono et al. 2018), in which a standard pyrethroid insecticide‐treated net (Olyset ITN) was compared with a novel net that combined pyrethroid with an insect growth regulator, pyriproxyfen (Olyset DUO net, also known as ITN‐PPF). The ITN‐PPF reduced the incidence of clinical malaria in children by 12% and vector numbers by 22%, compared to the standard ITN (Tiono et al. 2018). Our objective was to analyse population genetic data from *An. gambiae* samples collected during and after the trial to assess the impacts on: (1) the spatial genetic structure; (2) genetic diversity and inbreeding (as general, although imperfect (Buffalo 2021) proxies for population size); (3) putative signatures of selection in the genomes of mosquitoes collected from treated vs. control clusters (see Section 2 for more detailed justification). We provide a framework to detect changes in population structure in the context of field trials to give insight into the impacts of control on fragmentation and elimination of vector populations.

## 2 Methods

### 2.1 Study Design and Entomological Sample Collection

Briefly, the AvecNet stepwise cRCT was a two‐arm trial undertaken in 2014–2015 in Banfora, Burkina Faso (Tiono et al. 2018). In this area, malaria is endemic and largely transmitted by species of the morphologically identical *An. gambiae s.l*. complex (*An. arabiensis*, *An. gambiae*, and *An. coluzzii*). The mosquito population, and consequently malaria incidence, is highly seasonal, with peaks in abundance generally concentrated during the rainy season, which occurs between July and November.

**FIGURE 1:**
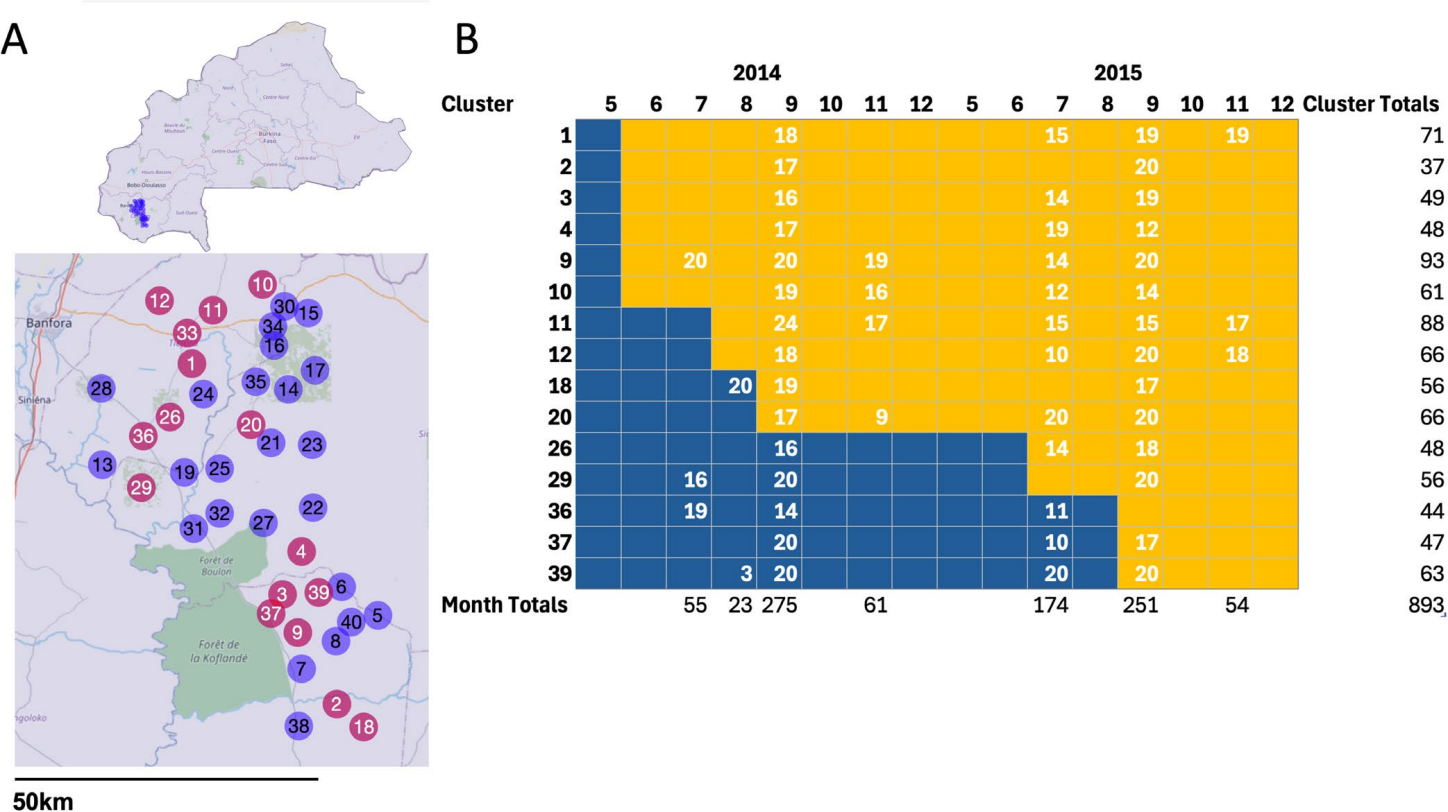
Clinical trial and collection setup. (A) Map of Burkina Faso highlighting the Banfora region (top panel) and a zoom in on the centroid of the 40 clusters enrolled in the cRCT (bottom panel). Maroon circles indicate clusters from which mosquitoes were sequenced. (B) Timeline of the implementation of ITN (blue, control) and ITN‐PPF bednets (yellow, treated) in each cluster, by year and month, for clusters where mosquitoes were sequenced. Numbers within a cell indicate the number of *An. gambiae* specimens that were sequenced in that particular cluster: Timepoint with the total numbers per cluster given in the right‐hand column.

The cRCT aimed to compare the effectiveness against clinical malaria of the Olyset DUO net (hereafter ITN‐PPF or treated), which in addition to the pyrethroid permethrin, contains pyriproxyfen, an insect growth regulator, to the standard Olyset pyrethroid‐only ITN (hereafter ITN or control), impregnated with permethrin only. The trial included 81 consenting villages that were grouped into 40 clusters, each including an average of 50 children under 5 years old. At the start of the malaria transmission season in June 2014, five randomly chosen clusters were provided with ITN‐PPF bednets, while the remaining clusters were given ITN bednets. Five clusters were randomly chosen for further ITN‐PPF delivery each month from July to November 2014 so that by the end of 2014, each trial arm had an equal number of clusters. In 2015, ITN‐PPF were distributed in a similar fashion starting in June 2015. By the end of the trial in December 2015, all clusters had the ITN‐PPF nets (See Figure 1 for more information on net and cluster distribution). During the cRCT, mosquitoes were collected using CDC Light Traps in the same six randomly chosen households per cluster, every 4 weeks between May and December in 2014 and May and November in 2015. All mosquitoes were identified using microscopy, and a random subset of approximately 30% of *An. gambiae s.l*. was typed by PCR (Tiono et al. 2018). Full details of the study protocol and collections are available in Tiono et al. (2018).

### 2.2 Sequencing

**FIGURE 2:**
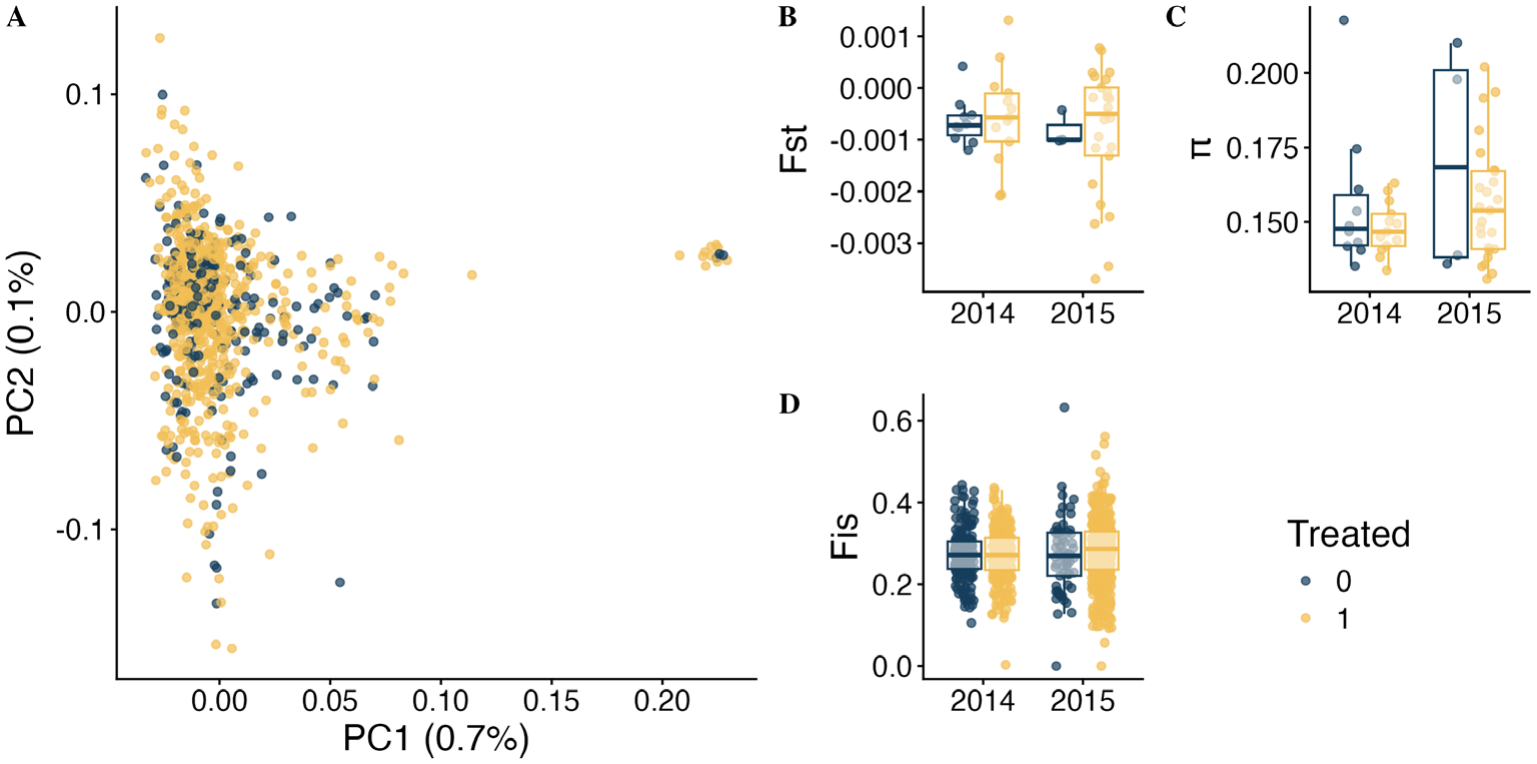
Analysis of spatial population structure and diversity. (A) Principal component analysis of *An. gambiae* samples, with variance explained by each PC in brackets. Panels B, C and D indicate between‐cluster: Timepoint *F*_ST_, within cluster: Timepoint π, and per‐individual *F*_IS_ by treatment and year, respectively. Yellow and blue points indicate control (0) and treated (1) sites and individuals, respectively.

A total of 16,785 mosquitoes were collected during this trial using CDC light traps, of which 14,489 (86%) were *An*. *gambiae sensu stricto* (hereafter *An. gambiae*). From these, we sequenced the whole genomes of 893 *An. gambiae*. We chose mosquitoes from clusters pre (ITN) and post‐treatment (ITN‐PPF) from around early (July/August 2014 and 2015), peak (September 2014 and 2015), and late (November 2014 and 2015) rainy seasons (the times of the year with the most available mosquito samples, facilitating as many between cluster/month comparisons as possible), while also ensuring we had good representation of space to capture the largest possible distances among clusters (Figure 2). To ensure sufficient sample sizes for population genetic analysis, we primarily restricted our choice to clusters × monthly timepoints for which > 10 samples were available (Lou et al. 2021). The clusters selected were an average of 29 km apart from each other (minimum of 3.53 km and maximum of 74.45 km). In total, we sequenced 240 *An. gambiae* specimens from ITN‐PPF clusters, and 174 from ITN clusters in 2014, and 418 and 61 *An. gambiae* sampled from ITN‐PPF and ITN clusters in 2015, respectively. Genomic DNA was extracted using a modified low salt Proteinase K buffer to maximise DNA yield (Korlević et al. 2021), and Illumina‐sequenced by Novogene UK. Mean/min/max per‐sample sequencing coverage was 3.41/1.04/15.01×, respectively, and mean/min/max % reads mapped per‐sample was 99.14/64.68/99.59% (for per‐sample sequencing data, see Table S1). % (for per‐sample sequencing data see Table S1). Samples were grouped for analysis by cluster and by year/month of collection. Each sample grouping is hereafter referred to as a cluster:timepoint.

### 2.3 Population Genetic Analyses

We investigated the impact of ITN‐PPF on the spatial genetic structure of *An. gambiae* during the study in multiple ways. First, we estimated kinship between all mosquito samples to build a dispersal kernel from the geographic distance between close kin (Sharma et al. 2022). This analysis aimed to determine whether kinship decayed significantly with geographic distance (i.e., displayed isolation‐by‐distance) and whether isolation by distance was greater between samples from treated/treated and treated/control vs. control/control clusters (Jasper et al. 2022; Filipović et al. 2020) (e.g., if dispersal occurred less frequently from treated sites). Second, we estimated *F*_ST_ between groups of samples from each cluster: timepoint (year‐month) and all other clusters: timepoints, to determine whether treated sites had higher mean genetic differentiation and, therefore, potential population fragmentation (Keven et al. 2024; Lamy et al. 2012) (possibly due to reduced dispersal or diversity in smaller populations). Finally, we performed a principal component analysis of genotype‐likelihoods from all mosquito samples to test whether those collected from treated (ITN‐PPF) clusters have more discrete genetic clusters than those from control (ITN) clusters (Keven et al. 2024; Lamy et al. 2012).

We investigated the impact of the ITN‐PPF intervention on the genetic diversity of *An. gambiae* using two metrics: nucleotide diversity (π) and individual inbreeding coefficient (*F*_IS_). We estimated *F*_IS_ per individual: an increase in per‐individual inbreeding is a result of mating between related individuals and of low genetic diversity in each cluster: timepoint. Finally, we performed genome‐wide scans of *F*_ST_ between treated and control sites to identify potential regions of the genome subject to selection in candidate insecticide‐resistance regions (Lucas et al. 2023). Almost all of the genome scan was in negative *F*_ST_, indicating greater genetic diversity within than between the compared populations. As such, we intersected the genomic coordinates of windows with positive *F*_ST_ values with the *An. gambiae* PEST genome annotation, downloaded from VectorBase (Giraldo‐Calderón et al. 2015), resulting in 49 genes (Table S2). The total sample size (893) and mean sequence coverage level (3.43)are sufficient to accurately estimate individual inbreeding coefficients (Vieira et al. 2013), kinship coefficients (Hanghøj et al. 2019), the site‐frequency spectrum for the entire sequenced population (for PCA), and for treatment/year subdivisions (for *F*_ST_ between years and treatments for genome scans) (Alex Buerkle and Gompert 2013). Sequenced sample sizes within cluster: timepoints ranged from 3 to 20, with sample sizes < 10 excluded from per‐cluster: timepoint‐based analysis, such as *F*_ST_, based on the expectation that low coverage sequencing of sample sizes between 10 and 20 can recover similar results as higher coverage and sample sizes (Lou et al. 2021).

## 3 Results

No discernible clustering pattern was present in an all‐sample PCA (Figure 2A), which revealed two groups along PC1 (Figure 2A) but these did not correspond to any specific cluster, timepoint, or treatment arm. Instead, the small group beyond the value of 0.2 of PC1 is from mosquitoes of a mix of clusters and timepoints (Figures S1 and S2) indicating possible cryptic population structure associated with currently unknown factors. We were also not able to detect any spatial structure and consequently no change in spatial structure in mosquito populations in association with the intervention. The mean *F*_ST_ between a single cluster: timepoint, and all other clusters: timepoints, was very low (mean *F*_ST_: −0.00073, 95% CI: −0.00109, 0.00013), with a negative *F*_ST_ value denoting greater genetic diversity within populations than between them. There were no statistically significant changes in mean differentiation (*F*_ST_) between treated clusters (−0.000748) compared to control clusters (−0.000680) and between 2015 (−0.000841) and 2014 (−0.000597) (Figure 2B). These results suggest that the mosquito population did not detectably fragment or change in structure as a result of treatment as the trial progressed. We found no obvious potential dispersal events. Kinship coefficient values between 0.125 and 0.5 indicate close‐kin relationships (Lucas et al. 2023; Manichaikul et al. 2010). We found the mean kinship coefficient for our data was −0.66 [min = −2.95; max: −0.26] suggesting no close‐kin relationships in any of the samples. Moreover, we found no significant change in between‐sample relatedness in pre‐ or post‐treatment clusters from 2014 or 2015.

The nucleotide diversity (π) of *An. gambiae* from treated and control clusters in 2014 and 2015 was between 0.13 and 0.21, with a mean of 0.15, comparable to previous estimates of West African *An. gambiae* (Miles et al. 2017; Anopheles gambiae 1000 Genomes Consortium 2020).

Median nucleotide diversity was slightly lower (though statistically insignificant) in *An. gambiae* from treated (0.154) vs. control (0.168) clusters in 2015 (Figure 2C). Similarly, the median inbreeding coefficient (*F*_IS_) was slightly higher (also statistically insignificant) in individuals from treated (0.287) vs. control (0.270) clusters in 2015 (Figure 2D). Finally, a genome scan of *F*_ST_ between treated and control clusters did not show any peaks of elevated differentiation in regions containing insecticide resistance genes between treated and control clusters (Figure S3, Table S2) characteristic of selection at resistance loci caused by vector control (Lucas et al. 2023). Taken together, the results indicate that the previously estimated 22% reduction in mosquito population density caused by the ITN‐PPF nets was not associated with alterations in the spatial genetic structure and diversity of *An. gambiae* in the trial region, or to produce signs of selection (linked to insecticide resistance, for example), during the time span of the trial.

## 4 Discussion

During the AvecNet cRCT, the ITN‐PPF nets produced a 12% reduction in clinical malaria in children and a 22% reduction in mosquito density (Tiono et al. 2018) compared to pyrethroid‐only ITNs. These epidemiological and entomological impacts did not coincide with detectable changes in mosquito genetic population structure between treated and control clusters, as seen by our analysis of whole genome sequence data of *An. gambiae* mosquitoes, densely sampled over a fine spatial scale. Our findings suggest that an epidemiological benefit from a successful vector control intervention is not necessarily associated with detectable changes in the vector population itself. To our knowledge, this is the first study reporting population genetic data, coupled with corresponding entomological and epidemiological endpoints in a cRCT setting.

Depending on the magnitude and modes of action of the intervention, epidemiological impacts may arise in the absence of substantial changes in vector population size and structure. For example, small reductions in mosquito life span might not necessarily impact recruitment or population size but could be sufficient to reduce the entomological inoculation rate (EIR) and consequently human infection risk (Smith et al. 2005; Grisales et al. 2021). Similarly, interventions that work by changing mosquito behaviour (e.g., impeding their ability to find or feed on a host) might substantially reduce human exposure to biting without reducing vector population size. For example, spatial repellents that impede vector ability to locate or feed on a human host might increase the time required for mosquitoes to find a blood meal and their probability of becoming infected (fewer host contacts), but may not diminish mosquito population size if other non‐human hosts are available to feed on (e.g., livestock). Another possibility is that variation in vectorial capacity due to genetic predisposition to *Plasmodium* infection (Pollitt et al. 2015; Blandin et al. 2004), or the presence of older mosquitoes that have taken infected blood meals from multiple hosts and contribute disproportionately to malaria transmission (Pollitt et al. 2015; Guelbéogo et al. 2018) means that interventions that preferentially target these high‐contribution mosquitoes could dramatically reduce malaria prevalence while leaving overall population structure largely intact. Such mechanisms reduce the EIR and cut malaria risk even in the absence of substantial vector population size reduction or local extinction.

In the case of the AvecNet trial, the additional sterilising effect of pyriproxyfen was sufficient to reduce mosquito population sizes by 22% and the likelihood of clinical malaria in children by 12% in the ITN‐PPF cohort without altering mosquito vector population structure or diversity.

Previously published work examining the impact of vector control on population genetic structure and diversity in concert with entomological or epidemiological indicators (albeit none in a cRCT setting) (Keven et al. 2024; Hetzel et al. 2017; Trape et al. 2014; Sougoufara et al. 2016), reveals no consistent pattern of entomological or epidemiological effect required to achieve a population genetic impact. In Dielmo, Senegal, ITN‐associated reductions of malaria prevalence from 86% to 0.3% over 22 years and near elimination of *An. gambiae* in favour of *An. Arabiensis* were not accompanied by any changes in diversity or inbreeding (Sougoufara et al. 2017). By contrast, ITN administration in Papua New Guinea was associated with changes in *An. hinesorum* and *An. farauti* spatial population structure, as well as reductions of 38% in caught *Anopheles* and declines in malaria transmission intensity of 11.1%–0.9% (Keven et al. 2024; Hetzel et al. 2017). It is possible that the reduction in vector population density reported in the ITN‐PPF clusters of the AvecNet trial, and any underlying transient impact on population allele frequencies, might not have been strong enough to be detectable in the face of homogenising gene flow. Even if ITN‐PPF nets caused temporary local extinction of malaria vectors in some clusters, recolonisation through migration might occur quickly enough to be indistinguishable from population persistence. *Anopheles* mosquitoes readily disperse 2‐3 km (Guerra et al. 2014) (though confident inference of dispersal is challenging due to low recapture rates and high heterogeneity in larval habitats), and sporadic long‐range wind‐borne dispersal has been observed in West Africa—far more than *Aedes* mosquitoes, which may move 10 s–100 s of metres in an individual lifespan (Huestis et al. 2019). Furthermore, genomic data from a wider scale show a relative lack of genetic structure over the span of West Africa (Anopheles gambiae 1000 Genomes Consortium 2020). Together, these data suggest that using population genetic data as an indicator of a successful vector control outcome may be of limited use when the impact of vector control on entomological indicators is modest. This might also be true in some cases where an intervention elicits large epidemiological and entomological impacts, but large mosquito population sizes and immigration from outside the trial area make it difficult to detect changes in vector population genetic analyses. In the latter case, application of our approach to situations where evaluations are conducted at a larger spatial scale than a typical cRCT (Gansané et al. 2022; Gonahasa et al. 2025; Maiteki‐Sebuguzi et al. 2023) may have more utility.

Population genetics data have been able to detect long‐term trends in vector populations brought about by vector control (e.g., by detecting genetic signals of population crashes in *An. gambiae* in Kenya (Miles et al. 2017)), hence, analysing data from longer‐term control programmes or trials with larger entomological effect sizes (Gonahasa et al. 2025; Mosha et al. 2024; Protopopoff et al. 2018), may yield stronger population genetic signals. Although the minimum number of samples per analysis cohort (e.g., treated/untreated or per cluster‐timepoint) was adequate, the step‐wedge design of the AvecNet trial (Figure 1) led to unequal sample sizes of treated and control samples in 2015 (Figure 1B, Figure 2). Trial designs that allow comparisons between larger treated and control cohorts throughout the trial (e.g., parallel‐group, platform, factorial) may allow more even sampling of treated vs. control populations as trials progress.

We show that changes to population structure are not necessarily associated with significant epidemiological or entomological impacts of vector control, but population genetic techniques may become more reliably useful when the goal of an intervention is to eliminate malaria vector populations rather than just reducing human exposure. At low population sizes, acquiring adequate numbers of mosquitoes to robustly estimate population size may be challenging, whereas a small number of specimens may be sufficient to elucidate genomic signatures of vector population decline (e.g., inbreeding, differentiation). As discussions progress around the deployment of gene‐drive technology, as well as other biological control tools that may eliminate mosquito vector populations (Bier 2022; Nolan 2020), it is also likely that genomics‐based approaches, such as those developed here for estimating mosquito population diversity, structure, and sizes will become crucial for measuring the impact of vector control.

## Conflicts of Interest

The authors declare no conflicts of interest.

## Supporting information


**Table S1:** Sample sequencing and collection data. Per‐sample (sample name) sequencing depth of coverage (mean_coverage), percentage of reads mapped to the *An. gambiae* PEST reference genome, collection date, collection month and year, village, location (latitude and longitude), collection method (method), result of species typing (species), trial cluster (cluster) and whether the sample was from a treated cluster/timepoint or not (treated, 0/1).
**Table S2:** Result of genome‐wide *F*_ST_ scan between treated and untreated samples. Rows denote genes located in windows of positive *F*_ST_, labelled by chromosome scaffold (chrom), the window midpoint (window_middle_position), the number of variable sites in the window (nsites), Hudson's *F*_ST_ (*F*_ST_), the type of annotation, the strand (strand), the gene name (VectorBase ID and description, name).

**Figure S1:** Principal component analysis (PCA) of sequenced *An. gambiae* from the AvecNet trial. Panels A, B and C indicate PCs 1/2, 3/4, and 5/6 respectively, indicated on the X and Y axes. Point colour indicates cluster.
**Figure S2:** Principal component analysis (PCA) of sequenced *An. gambiae* from the AvecNet trial. Panels A, B and C indicate PCs 1/2, 3/4, and 5/6 respectively, indicated on the X and Y axes. Point colour indicates Year:Month.
**Figure S3:** Genome‐wide *F*_ST_ between treated and untreated samples. X axis indicates chromosomal position, Y axis indicates *F*_ST_ in 50 Kb windows. Plot is faceted by chromosome.

## Data Availability

Analysis code and bioinformatic workflows are available at http://github.com/tristanpwdennis/avecnet_popgen. Raw reads have been deposited in the European Nucleotide Archive under accession PRJEB62110.
