# Supplementary material for: Must Epidemiologically Impactful Vector Control Interventions Disrupt Mosquito Population Structure? A Case Study of a Cluster‐Randomised Controlled Trial
Source: Evol Appl. 2025 Oct 27;18(11):e70173. doi: 10.1111/eva.70173 (PMC12558597; doi:10.1111/eva.70173)
Supplement: Supplementary file 2 — Figure S1: Principal component analysis (PCA) of sequenced An. gambiae from the AvecNet trial. Panels A, B and C indicate PCs 1/2, 3/4, and 5/6 respectively, indicated on the X and Y axes. Point colour indicates cluster. Figure S2: Principal component analysis (PCA) of sequenced An. gambiae from the AvecNet trial. Panels A, B and C indicate PCs 1/2, 3/4, and 5/6 respectively, indicated on the X and Y axes. Point colour indicates Year:Month. Figure S3: Genome‐wide F ST between treated and untreated samples. X axis indicates chromosomal position, Y axis indicates F ST in 50 Kb windows. Plot is faceted by chromosome. [file EVA-18-e70173-s002.docx]

*Supplementary Methods*

We sequenced the whole genomes of 893 *An. gambiae s.s.* to a median depth-of-coverage of 3.58X with 2x150bp paired-end Illumina reads. Raw reads were trimmed using fastp(Chen et al. 2018), and aligned to the *An. gambiae* PEST reference genome(Holt et al. 2002), using bwa-mem(Li and Durbin 2009). Alignments were sorted, with duplicates marked, using samtools(Danecek et al. 2021). BAM alignments were grouped into cohorts for analysis, consisting of all 893 samples, and samples grouped by cluster and timepoint (year-month, see **Figure 1** for cohort sizes). Genotype-likelihoods (GLs) and site allele frequencies (SAFs) were inferred using ANGSD(Korneliussen et al. 2014). All-sample GL calling was performed for principal-component analysis (PCA) and inbreeding coefficient (Fis) estimation. Per- cluster per-timepoint site allele frequency estimation (see **Figure 1 for cohort sizes**) was performed on the whole genome for the estimation of per-cluster per-timepoint *F_ST_* and π. For the all-sample callsets, GLs were called with the reference genome as the major allele (-doMajorMinor 4), filtering GLs based on a a minor allele (maf) frequency of 0.01 (-minmaf 0.01), maximum SNP p value 0.05 (-snp pval 0.05), covered only by properly paired reads (-only proper pairs 1), and uniquely mapped reads with a mapping quality of 20 (-uniqueOnly 1 -minMapQ 20). For the per-cluster per-timepoint SAF estimation, we used the parameters above, but omitted depth, SNP p-value, and maf filters. Filtering that biases the SFS towards more frequent sites can substantially distort estimates of *F_ST_* and π. Full parameters are detailed in the avecnet_popgen repository detailed in the main text, under bin/angsd scripts. Principal component analysis and per-sample Fis estimation was performed on chromo- some arm 3L GLs using PCAngsd(Meisner and Albrechtsen 2018). Folded site-frequency spectra (SFS) were calculated from SAF files for all samples, as well as per-cluster and per-timepoint, using realSFS(Korneliussen et al. 2014). For estimating differentiation of each cluster and timepoint with respect to the wider metapopulation, two-dimensional(2d)-SFS were generated between the allsample SFS, and per-cluster per-timepoint SFS. Each 2d-SFS was used to infer Hudson’s *F_ST_* between each pair of cluster:timepoints. Hudson’s *F_ST_* was chosen as it is more robust to sample size differences between populations(Bhatia et al. 2013). Per-cluster/timepoint SFS were used to estimate per-population π. PCA, *F_ST_*, π, and *F_IS_* data were analysed in R v4.2.2(R Core Team, 2006). GLMs assessing the effects of treatment and year on *F_ST_*, π, and Fis were performed using glm. Negative binomial GLMMs were performed using lme4(Bates et al. 2015). Residuals were examined using DHARMa v0.4.6(Hartig 2022). Plotting was performed with ggplot2(Wickham 2016) library. To assess the effect of DUO on per-individual *F_IS_*, and per-cluster-, per-timepoint *F_ST_*, and π, Gaussian GLMs using the three parameters prior as a function of DUO administration and time, were parameterised. All three GLMs returned insignificant results.

*Supplementary Figures*

**
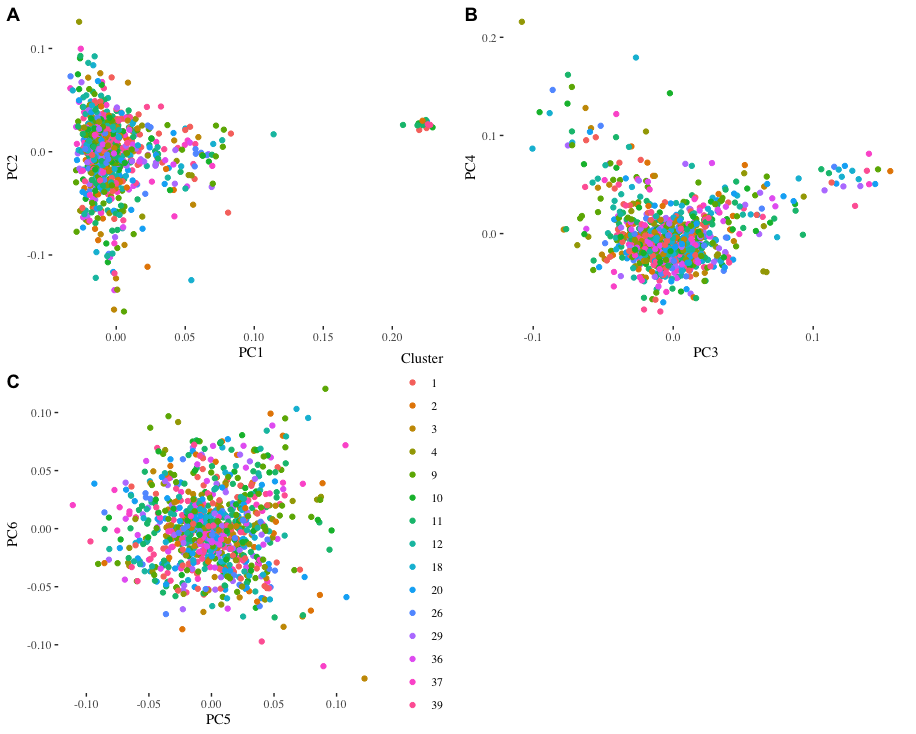
**

**Figure S1:** Principal component analysis (PCA) of sequenced *An. gambiae* from the AvecNet trial. Panels A, B and C indicate PCs 1/2, 3/4 , and 5/6 respectively, indicated on the X and Y axes. Point colour indicates cluster.


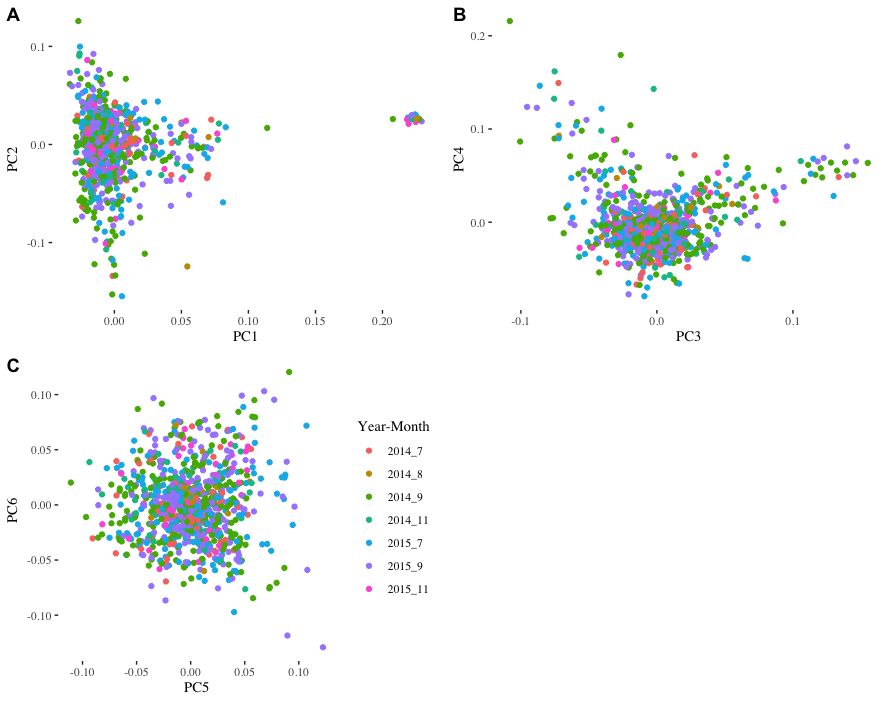


**Figure S2:** Principal component analysis (PCA) of sequenced *An. gambiae* from the AvecNet trial. Panels A, B and C indicate PCs 1/2, 3/4 , and 5/6 respectively, indicated on the X and Y axes. Point colour indicates Year:Month.

**
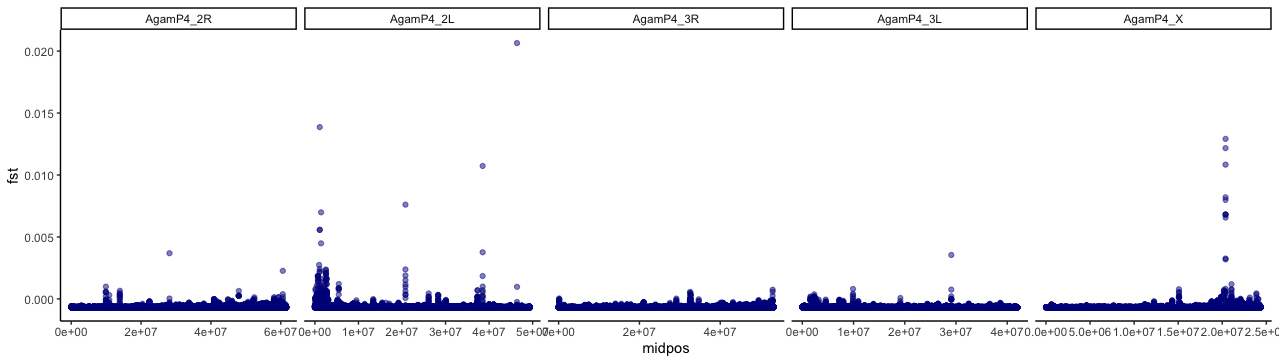
**

**Figure S3**: Genome-wide *Fst* between treated and untreated samples. X axis indicates chromosomal position, Y axis indicates *Fst* in 50Kb windows. Plot is faceted by chromosome

Bates, Douglas, Martin Mächler, Ben Bolker, and Steve Walker. 2015. ‘Fitting Linear Mixed-Effects Models Using Lme4’. *Journal of Statistical Software* 67 (October): 1–48. https://doi.org/10.18637/jss.v067.i01.

Bhatia, Gaurav, Nick Patterson, Sriram Sankararaman, and Alkes L. Price. 2013. ‘Estimating and Interpreting FST: The Impact of Rare Variants’. *Genome Research* 23 (9): 1514–21. https://doi.org/10.1101/gr.154831.113.

Chen, Shifu, Yanqing Zhou, Yaru Chen, and Jia Gu. 2018. ‘Fastp: An Ultra-Fast All-in-One FASTQ Preprocessor’. *Bioinformatics* 34 (17): i884–90. https://doi.org/10.1093/bioinformatics/bty560.

Danecek, Petr, James K Bonfield, Jennifer Liddle, et al. 2021. ‘Twelve Years of SAMtools and BCFtools’. *GigaScience* 10 (2): giab008. https://doi.org/10.1093/gigascience/giab008.

Hartig, Florian. 2022. *DHARMa: Residual Diagnostics for Hierarchical (Multi-Level/Mixed) Regression Models*. Released. https://cran.r-project.org/web/packages/DHARMa/vignettes/DHARMa.html.

Holt, Robert A., G. Mani Subramanian, Aaron Halpern, et al. 2002. ‘The Genome Sequence of the Malaria Mosquito Anopheles Gambiae’. *Science* 298 (5591): 129–49. https://doi.org/10.1126/science.1076181.

Korneliussen, Thorfinn Sand, Anders Albrechtsen, and Rasmus Nielsen. 2014. ‘ANGSD: Analysis of Next Generation Sequencing Data’. *BMC Bioinformatics* 15 (1): 356. https://doi.org/10.1186/s12859-014-0356-4.

Li, Heng, and Richard Durbin. 2009. ‘Fast and Accurate Short Read Alignment with Burrows–Wheeler Transform’. *Bioinformatics* 25 (14): 1754–60. https://doi.org/10.1093/bioinformatics/btp324.

Meisner, Jonas, and Anders Albrechtsen. 2018. ‘Inferring Population Structure and Admixture Proportions in Low-Depth NGS Data’. *Genetics* 210 (2): 719–31. https://doi.org/10.1534/genetics.118.301336.

R Core Team. n.d. ‘R: A Language and Environment for Statistical Computing. R Foundation for Statistical Computing, Vienna, Austria.’ Methodology Reference. Accessed 3 May 2023. https://www.eea.europa.eu/data-and-maps/indicators/oxygen-consuming-substances-in-rivers/r-development-core-team-2006.

Wickham, Hadley. 2016. *Create Elegant Data Visualisations Using the Grammar of Graphics*. Springer-Verlag New York. https://ggplot2.tidyverse.org/.
